# Science and innovation in malaria diagnostics

**DOI:** 10.1186/1475-2875-13-S1-O19

**Published:** 2014-09-22

**Authors:** Sanjeev Krishna

**Affiliations:** 1 St. George’s, University of London, UK

## 

Five species of *Plasmodium* naturally infect humans. Some species cause non-specific symptoms that can sometimes progress rapidly to severe and fatal outcomes. Early diagnosis and appropriate treatment interrupts progression and cures disease. Confirmation of diagnosis of malaria currently relies on microscopy, or on application of rapid diagnostic tests (RDTs) that are becoming increasingly widely available and are recommended to confirm infection before treating it. The diagnosis of malaria by itself is not sufficient to optimise individual therapies because there is a growing problem of multidrug resistance in parasites (particularly *Plasmodium falciparum*). This limits the use of some drugs or combinations by severely compromising their efficacy.

Diagnostic strategies for management of malaria can therefore be improved in several ways. First by an increase in sensitivity of detection of parasites that should improve of the thresholds for detection by the current generation of RDTs because they cannot identify low parasitaemias. Second, a diagnostic that can differentiate all the naturally infecting species of parasite needs to be developed. Finally, rapid (point-of-care) assessment of the drug resistance status of parasites will add greatly to the treatment strategies available to manage individual patients. Technological platforms that can deliver information that is currently missing for the personalised management of malaria infections are being developed and these advances will be presented and discussed in the context of the global burden of disease caused by malaria.

